# The Oscillating Potential Model of Visually Induced Vection

**DOI:** 10.1177/2041669517742176

**Published:** 2017-11-24

**Authors:** Takeharu Seno, Ken-ichi Sawai, Hidetoshi Kanaya, Toshihiro Wakebe, Masaki Ogawa, Yoshitaka Fujii, Stephen Palmisano

**Affiliations:** Faculty of Design, 12826 Kyushu University, Minami-ku, Fukuoka, Japan; Graduate Schools for Law and Politics, The University of Tokyo, Bunkyo-ku, Tokyo, Japan; Faculty of Human Informatics, Aichi Shukutoku University, Nagakute-shi, Aichi, Japan; Faculty of Human Relations, Fukuoka Jo Gakuin University, Minami-ku, Fukuoka, Japan; Faculty of Design, Kyushu University, Minami-ku, Fukuoka, Japan; Research Organization of OIC, Ritsumeikan University, Ibaraki, Osaka, Japan; University of Wollongong, Wollongong, NSW, Australia

**Keywords:** vection, latency, duration, magnitude, index, model

## Abstract

Visually induced illusions of self-motion are often referred to as *vection*. This article developed and tested a model of responding to visually induced vection. We first constructed a mathematical model based on well-documented characteristics of vection and human behavioral responses to this illusion. We then conducted 10,000 virtual trial simulations using this *Oscillating Potential Vection Model* (OPVM)*.* OPVM was used to generate simulated vection onset, duration, and magnitude responses for each of these trials. Finally, we compared the properties of OPVM’s simulated vection responses with real responses obtained in seven different laboratory-based vection experiments. The OPVM output was found to compare favorably with the empirically obtained vection data.

## Introduction

When a large area of the visual field is stimulated by coherent motion, stationary observers often (illusorily and incorrectly) perceive that they themselves are moving (typically in the opposite direction to the stimulus motion). This type of visually induced illusion of self-motion has traditionally been referred to as “vection” (e.g., Dichgans & Brandt, 1978; however, see Palmisano, Allison, Schira, & Barry, 2015, for other alternative uses of this term in the context of self-motion perception). While there are considerably earlier documented observations of vection,^1^ the first systematic experimental examinations of this phenomenon only appeared in print in the early 1970s. Following Brandt, Dichgans, and Koenig’s (1973) seminal paper, hundreds of vection studies have since been published. A recent PubMed search for the term *vection* produced 358 papers and book chapters (search conducted on the 19th August, 2017). Of these vection articles, there have also been several major reviews of this literature, including comprehensive reviews of the early vection research (see Dichgans & Brandt, 1978; Howard, 1982), as well as later reviews of more recent vection developments (e.g., Hettinger, Schmidt, Jones, & Keshavarz, 2014; Palmisano, Allison, Kim, & Bonato, 2011; Palmisano et al., 2015; Riecke, 2010; Väljamäe, 2009).

Due to recent improvements in virtual reality and self-motion simulation technology, vection is now becoming an increasingly popular topic of research. For example, one particularly active area of vection research is the investigation of potential relationships between visually induced vection and visually induced motion sickness (e.g., D’Amour, Bos, & Keshavarz, 2017; see Keshavarz, Riecke, Hettinger, & Campos, 2015, for a review). However, despite the recent upsurge in scientific research on vection, there have been comparatively few attempts to mathematically model the phenomenon itself (see Jürgens, Kliegl, Kassubek, & Becker, 2016; Zacharias & Young, 1981, for two exceptions; both models were focused on explaining the onset latency of visually induced illusions of self-rotation, known as circular vection). This article seeks to remedy this situation by developing a mathematical model of how observers respond to visually induced vection. Specifically, this model is aimed at explaining how the (objective) state of vection might be translated into the observer’s (subjective) vection ratings and other reporting behaviors. Thus, the model must be able to capture both the reported characteristics of the vection time course (its reported onset latency, its reported duration, the occurrence of reported dropouts, etc.) as well as the key aspects of its reported subjective experience (such as its reported strength or intensity).

While vision is not the only modality that can induce illusions of self-motion (see also auditory, haptokinetic, arthokinetic, and biomechanical vection),^2^ the majority of studies conducted to date have investigated visually induced self-motion (see Palmisano et al., 2015, for a recent review). Traditionally, this “visual” vection research has examined how different visual stimulus parameters affect the onset, strength, and speed of the vection experience (see Riecke, 2010). However, more recent research has also begun to examine how visually induced vection is influenced by cognitive factors (e.g., Lepecq, Giannopulu, & Baudonniere, 1995; Riecke, Schulte-Pelkum, Avraamides, von der Heyde, & Bülthoff, 2006) and the simultaneous stimulation of the nonvisual self-motion senses (e.g., Keshavarz, Hettinger, Vena, & Campos, 2014; Riecke, Väljamäe, & Schulte-Pelkum, 2009). Since we currently have a much greater understanding of visually induced vection, this article focusses specifically on developing a mathematical model for visually induced vection. From this point on in the article, we will refer to “visually induced vection” simply as “vection.” As the few past models have focused primarily on circular vection, we have instead chosen to focus on linear vection in the present article—as research on illusory self-translation has increased dramatically in recent years with the use of computer-generated vection studies. Accordingly, the model will be developed based on well-documented observations about responses to linear vection, and then tested using empirical response data obtained in seven recent experimental studies examining different types of linear vection.

In past studies, three characteristics of vection responding have been repeatedly observed: (a) there is a finite delay of 1 to 10 s after display motion begins before vection onset is first reported, (b) there is then an increase in reported vection strength over time until reported vection strength eventually plateaus, and (c) vection dropouts are often reported after the initial vection induction and before the display motion ceases (e.g., Dichgans & Brandt, 1978; Howard, 1982; Riecke, 2010). As the vection-inducing motion stimuli used in these studies typically had constant speeds and were presented continuously, these characteristics of human vection responding suggest that subjective experiences of vection are both unstable and oscillatory. The past research also indicates that there can be substantial individual differences in vection responding to the same inducing stimulus (in terms of both reported vection strength and reported vection time course). Thus, we aimed to incorporate all of these aspects of human vection responding into our mathematical model.

In principle, there are many potential benefits of creating a viable model of vection responding. Using such a model, large numbers of conditions can be investigated as the outputs of millions of simulated trials can be generated easily. By varying the internal parameters of our model, we aimed to simulate the substantial individual differences in human responding observed in past vection experiments. If successful, these model simulations should reveal important insights into (a) the origins of these individual differences in reporting or responding and (b) the processes or mechanisms involved in both consciously experiencing and responding to vection. This in turn should suggest new future directions for human vection research. In the past, the construction of mathematical models for vision science has led to increased research activity into, and improved knowledge of, many different types of perceptual phenomena (such as motion perception and surface perception—see Adelson & Bergen, 1985; Motoyoshi, Nishida, Sharan, & Adelson, 2007).

This article will focus on developing a mathematical model of responding to visually induced vection: The Oscillating Potential Vection Model (OPVM). There are no specific sensory input variables included in the model. The model instead includes a general parameter representing the inducing potential of the optic flow. The focus of the model is therefore on generating the following three vection response outputs (similar to those obtained in most typical laboratory experiments) based on this simulated inducing stimulus: (a) vection onset latency, (b) simulated vection duration, and (c) vection strength. To investigate the limitations of the model and to further refine it, we also tested our model by comparing the simulated onset latency, duration, and magnitude responses generated by the model to equivalent responses obtained from human observers in real vection experiments.

## The OPVM

This model investigates the processes underlying vection responding to visual motion stimulation. Vection is typically induced by large patterns of optical flow. However, the model is not focused on relationships between physical modulations of this optic flow and the objective experience of vection, but instead it focusses on how the reported subjective experience of vection might be generated.

### Constructing the Mathematical Model

The mathematical model was constructed to conform to the following well-documented properties of vection responding:

*Property 1:* There will always be a finite delay between the start of the visual motion stimulation and the onset of vection (e.g., Brandt et al., 1973; Bubka, Bonato, & Palmisano, 2008; Dichgans & Brandt, 1978). During this initial period in the trial, the observer typically perceives the optic flow as being entirely due to object motion. Vection onset latency is thought by many to represent the time it takes to resolve sensory conflicts generated by presenting optic flow displays to physically stationary observers (Jürgens et al., 2016; Mergner, Schweigart, Müller, Hlavacka, & Becker, 2000; Palmisano et al., 2011; Weech & Troje, 2017; Zacharias & Young, 1981). Since the vestibular stimulation which would normally accompany this type of visual self-motion information is absent, this visual-vestibular conflict is proposed to cause the observed delay between the start of visual motion stimulation and the first report of vection. However, this vection onset latency might also represent the time it takes to suppress the default visual processing responsible for object motion perception, prior to the actual induction of vection (e.g., Palmisano, Barry, De Blasio & Fogarty, 2016).

*Property 2:* After the initial onset of vection, the observer first perceives a mixture of object-and-self-motion before he or she eventually experiences exclusive self-motion (known as vection saturation—see Dichgans & Brandt, 1978). As a result, vection magnitude generally builds toward a plateau over the course of the trial (e.g., Apthorp & Palmisano, 2014).

*Property 3:* Vection can “dropout” after induction—particularly when the induced vection is weak or ambiguous. It is common in these situations for the observer to experience a perceptual alternation between vection (ON) and nonvection (OFF) periods (e.g., Brandt et al., 1973; Kano, 1991; Nakamura, 2010; Seno, Ito, & Sunaga, 2009).

Any model of vection responding must therefore be capable of simulating both supra- and subthreshold vection experiences during continuous periods of visual motion stimulation. Accordingly, OPVM includes a threshold (θ) that demarks ON and OFF vection periods (ON periods occur whenever the modeled response exceeds the threshold for reporting a conscious experience vection).

Vection response output at time *t* during the trial (i.e., V(t)) is described by the following formula (with 1 and 0 representing ON and OFF vection periods, respectively):

$$V(t)=\{\begin{array}{c} 1\text{if}v(t)\geq \theta \\ 0\text{otherwise} \end{array}$$

where v(t) describes internal state regarding the potential for the participant to experience vection, and θ is the threshold for reporting a conscious experience of vection.

We also employed a function $v_{\text{trend}}(t)$ that increased this potential gradually over time (to satisfy aforementioned Properties 1 and 2), as well as a periodic function $v_{\text{oscil}}(t)$ (to satisfy aforementioned Property 3):

$$v(t)=v_{\text{trend}}(t)\cdot v_{\text{oscil}}(t)$$

Directly after the start of the stimulus motion (t=0), the vection response output V(t) should be 0 (i.e., only object motion should be perceived at this time). v(t) should then increase over time, eventually starting to plateau following expected vection saturation. Thus, we set the function $v_{\text{trend}}(t)$ as follows:

$$v_{\text{trend}}(t)=1-\text{exp}(-\alpha t)(\alpha >0)$$

where the parameter α controls the latency to vection onset through $v_{\text{trend}}(t)$. α depends on visual motion stimulation *S* (which is regarded as the inducing potential of the optic flow). When there is no visual motion stimulation (S=0), then α(0) will be 0. However, when a vection inducing motion stimulus is presented (S>0), then α(S)>0. *S* and α(S) both increase with the vection inducing potential of the optic flow (e.g., they should increase as the size of the optic flow pattern increases—Dichgans & Brandt, 1978). As α(S) increases, the acceleration rate of $v_{\text{trend}}$ also increases, which should result in shorter onsets and stronger magnitudes of vection responding. This is how the relationship between the visual motion stimulation and vection responding was incorporated into the model.

To model the alternation between ON and OFF vection reporting periods, we used a simple sinusoidal function:

$$v_{\text{oscil}}(t)=\frac{\text{sin}(2\pi t/T)+\beta }{1+\beta }(\beta >1)$$

where *T* is the period of the oscillation during the plateau phase and β controls the value of the oscillation center. To perceive optic flow as being due to self-motion, it has been speculated that the system would need to first inhibit the default visual processing which is responsible for normally perceiving object or scene motion (e.g., Palmisano, Barry, De Blasio & Fogarty, 2016). The β and *T* values in this model control the oscillation between ON and OFF vection responding. It was proposed that they might represent the degree of inhibition of object motion processing (so as to instead favor self-motion processing) and the amount of time such inhibition is successful over the course of the “trial,” respectively.

**Figure 1.**
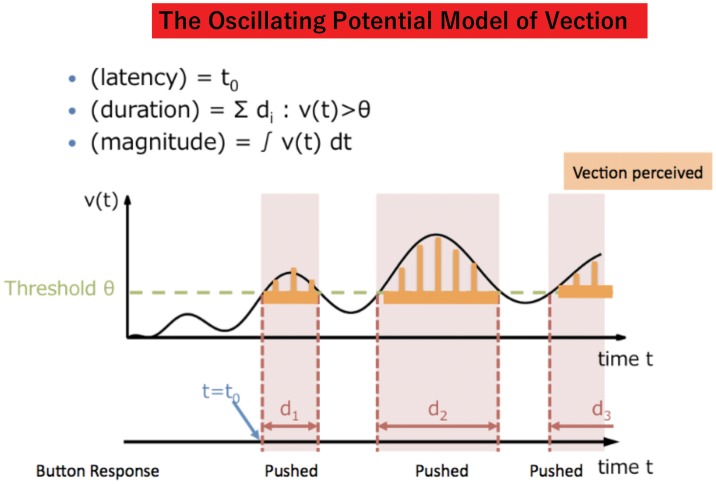
The Oscillating Potential Vection Model (OPVM). The horizontal black arrows indicate “TIME” from the stimulus onset. The black fluctuating sinusoidally curved line indicates the simulated internal state of the participant over time. Whenever this curved line exceeds the threshold indicated by the green dashed line, vection will be reported (the onset of reported vection can therefore be estimated as the first time the curved line cuts the threshold). The tan boxes in the figure indicate “with vection periods.” Thus, the total size of these saw-toothed tan areas can be converted into an estimate of the overall vection magnitude for the trial.

Our mathematical model of vection (OPVM) was thus created by multiplying these two functions, $v_{\text{trend}}(t)$ and $v_{\text{oscil}}(t)$. An example of the behavior of this model can be seen in Figure 1. The equation is able to satisfy all three of the vection properties identified previously in Constructing the Mathematical Model section. Values of $v_{\text{trend}}(t)\text{and}v_{\text{oscil}}(t)$ can range from 0 to 1. The internal potential v(t) is therefore also able to vary from 0 to 1.

### Modeling Empirically Observed Individual Differences

**Figure 2.**
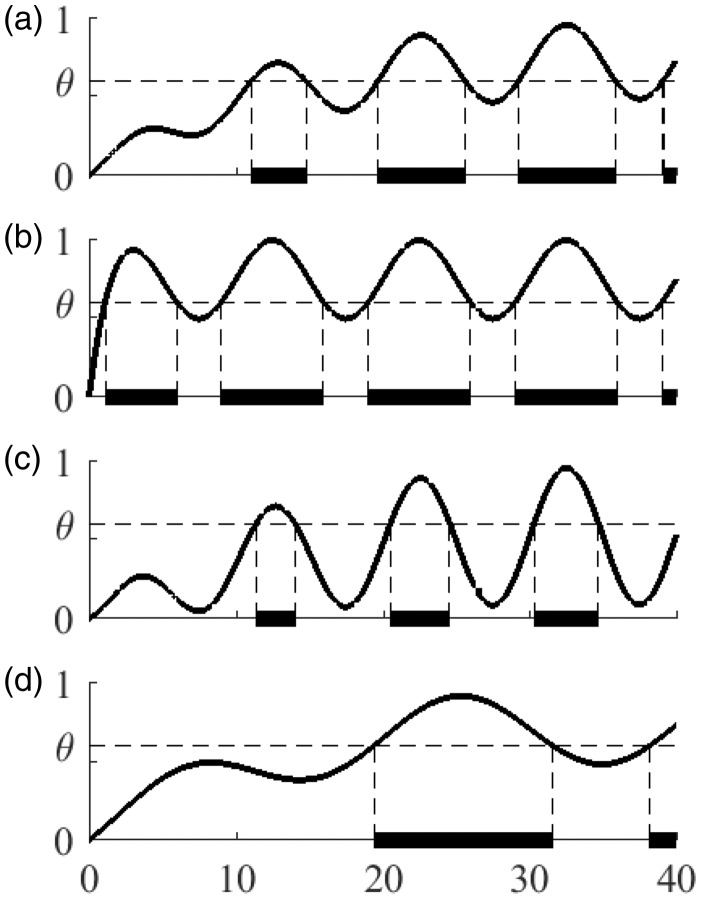
Examples of OPVM behavior with different α, β, *T*, and θ parameter sets. The horizontal and vertical axes are time and the value of v(t), respectively. The simulated participant experiences vection when v(t)>θ (indicated by bold lines on the horizontal axes). Please see the main text for descriptions of a)-d) above.

In this section, we will introduce and explain our mathematical model. Each parameter involved in the model (α, β, *T*, and θ) is important in determining the subjective vection response output (V(t)). Figure 2(a) shows the simulated internal potential and resulting vection response output when the values of α, β, *T*, and θ are 0.1, 3, 10, and 0.6, respectively. As αincreases from 0.1 to 1 in Figure 2(b), the simulated vection onset latency can be seen to decrease. When β is reduced from 3 to 1.2 in Figure 2(c), the duration of the ON periods are reduced (e.g., compared with Figure 2(a)). The value of *T* can also be seen to affect the duration of ON and OFF periods. As *T* increases from 10 to 20, the initial ON duration increases and the frequency of switching between ON and OFF periods decreases (see Figure 2(d)). By modifying the values of these parameters, it should therefore be possible to model empirically observed individual differences in human vection responding.

### Choosing the Vection Indices to Model

**Figure 3.**
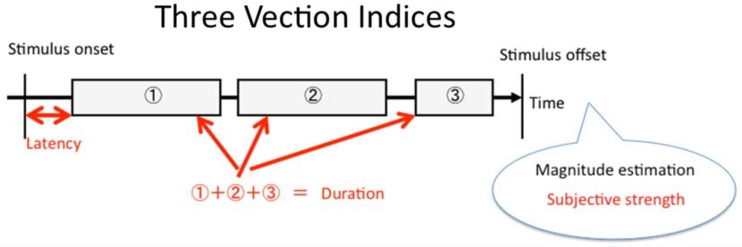
A schematic illustration of the three vection measures: latency, duration, and magnitude. The horizontal black arrow indicates “TIME” between the onset and offset of the stimulus presentation. Boxes 1, 2, and 3 indicate “with vection periods” and the spaces between them indicate “vection dropouts”.

We next used OPVM response output to reconstruct the typical vection indices obtained in human laboratory experiments. In the past, the three most commonly employed measures of vection^3^ obtained in such studies have been (a) the *latency to vection onset* (i.e., the delay between the start of the visual motion stimulation and the observer’s first reported experience of illusory self-motion), (b) the *total duration of the vection* (i.e., the total amount of time that the observer reported experiencing vection during the trial), and (c) magnitude estimates or ratings of the vection experience (e.g., verbal ratings using a linear scale from 0 = *no vection* to 100 = *very strong vection*) (see Figure 3).

In our review of the recent literature, we found more than 50 papers where all three of these measures were obtained in the same experiment (see Allison, Ash, & Palmisano, 2014; Apthorp & Palmisano, 2014; Bonato & Bubka, 2006; Bonato, Bubka, Palmisano, Phillip, & Moreno, 2008; Brandt et al., 1973; Bubka & Bonato, 2010; Bubka et al., 2008; Gurnsey, Fleet, & Potechin, 1998; Guterman, Allison, Palmisano, & Zacher, 2012; Keshavarz et al., 2015; Keshavarz, Speck, Haycock, & Berti, 2017; Kim & Palmisano, 2008, 2010a; Mohler, Thompson, Riecke, & Bülthoff, 2005; Nakamura, 2006, 2010, 2012, 2013a, 2013b, 2013c, 2013d, 2013e; Nakamura, Palmisano, & Kim, 2016; Nakamura, Seno, Ito, & Sunaga, 2010, 2013; Nakamura & Shimojo, 1998, 1999, 2003; Ogawa, Ito, & Seno, 2015; Ogawa & Seno, 2014; Ogawa, Seno, Matsumori, & Higuchi, 2015; Palmisano, 1996; Palmisano, Burke, & Allison, 2003; Palmisano & Chan, 2004; Palmisano, Gillam, & Blackburn, 2000; Palmisano & Kim, 2009; Palmisano et al., 2011, 2015; Palmisano, Summersby, Davies & Kim, 2016; Riecke et al., 2006, 2009; Riecke, Feuereissen, Rieser, & McNamara, 2011; Sasaki, Seno, Yamada, & Miura, 2012; Seno, Abe, & Kiyokawa, 2013; Seno & Fukuda, 2012; Seno, Funatsu, & Palmisano, 2013; Seno, Ito, & Sunaga, 2009, 2010, 2011; Seno, Ito, Sunaga, & Palmisano, 2012; Seno, Kitaoka, & Palmisano, 2013; Seno & Palmisano, 2012; Seno, Palmisano, & Ito, 2011; Seno, Palmisano, Ito, & Sunaga, 2012; Shirai, Imura, Tamura, & Seno, 2014; Shirai, Seno, & Morohashi, 2012; Tamada & Seno, 2015; Tarita-Nistor, González, Spigelman, & Steinbach, 2006).

While other studies did not obtained all three measures together, most obtained at least one or more of them^4^ (e.g., Allison, Zacher, Kirollos, Guterman, & Palmisano, 2012; Andersen & Braunstein, 1985; Ash & Palmisano, 2012; Ash, Palmisano, Apthorp, & Allison, 2013; Ash, Palmisano, Govan, & Kim, 2011; Ash, Palmisano, & Kim, 2011; Becker, Raab, & Jürgens, 2002; Brandt, Dichgans, & Büchele, 1974; Brandt, Wist, & Dichgans, 1975; Delorme & Martin, 1986; Diels, Ukai, & Howarth, 2007; Fushiki, Takata, & Watanabe, 2000; Giannopulu & Lepecq, 1998; Haibach, Slobounov, & Newell, 2009; Held, Dichgans, & Bauer, 1975; Howard & Heckmann, 1989; IJsselsteijn, de Ridder, Freeman, Avons, & Bouwhuis, 2001; Ishida, Fushiki, Nishida, & Watanabe, 2008; Ito & Shibata, 2005; Ito & Takano, 2004; Ji, So, & Cheung, 2009; Jürgens et al., 2016; Kano, 1991; Kennedy, Hettinger, Harm, Ordy, & Dunlap, 1996; Kim & Khuu, 2014; Kim, Palmisano, & Bonato, 2012; Lubeck, Bos, & Stins, 2015; Ohmi & Howard, 1988; Ohmi, Howard, & Landolt, 1987; Palmisano, 2002; Palmisano, Allison, & Howard, 2006; Palmisano, Apthorp, Seno, & Stapley, 2014; Palmisano, Bonato, Bubka, & Folder, 2007; Palmisano, Kim, & Freeman, 2012; Palmisano, Mursic, & Kim, 2017; Post, 1988; Previc & Donnelly, 1993; Riecke & Feuereissen, 2012; Riecke, Freiberg, & Grechkin, 2015; Riecke & Jordan, 2015; Seno, Palmisano, Ito, & Sunaga, 2013; Seno, Palmisano, Riecke, & Nakamura, 2015; Tanahashi, Ujike, & Ukai, 2012; Tarita-Nistor, González, Markowitz, Lillakas, & Steinbach, 2008; Telford & Frost, 1993; Telford, Spratley, & Frost, 1992; Thurrell & Bronstein, 2002; Wong & Frost, 1981).

In these laboratory studies, the human observers were exposed to patterns of optic flow. Then, they typically had to press a button when they first experienced vection and hold this button down as long as this experience continued (releasing the button if the vection “dropped out” and pressing it again if the experience returned). The observers would then also typically provide a magnitude rating of the vection experience for that trial after the display motion had ceased.^5^

### Reconstructing Vection Onset, Duration, and Magnitude From OPVM Response Output

We next used the OPVM response output to reconstruct each of these vection measures (onset, duration, and magnitude). Since a conscious vection experience occurs whenever v(t)≥θ, the first instance of v(t)≥θ in a simulation trial was used as the onset of vection. The total duration of vection was then calculated by summing all of the times in the particular simulation trial when v(t)≥θ. And finally, an estimate of the vection magnitude for the trial was calculated by integrating the area that the v(t) function covers above the threshold line.

## Testing OPVM

To investigate OPVM, we next conducted a large-scale virtual vection experiment. The three simulated response measures (onset latency, duration, and magnitude) were generated for each of the trials in this virtual experiment. Afterward, we compared this simulated response data with real data obtained previously in seven different vection experiments.

### Virtual Vection Experiment

OPVM was used to simulate a virtual vection experiment consisting of 10,000 trials. The aim was to generate vection onset, duration, and magnitude response data which displayed individual differences similar to those commonly seen in human participants (i.e., due to their different sensitivities to vection, different response biases, etc.). To this end, the values of each parameter used in the simulation were randomly drawn from uniform distributions—except for α, which was drawn from a log-uniform distribution for each virtual trial. These uniform (other than α) and log-uniform (in the case of α) distributions ranged between 0.01 and 1 for α, 0 and 5 for β, 3 and 20 for *T*, and 0 and 1 for θ. While θ was free to span all possible values between 0 and 1, the ranges of α and *T* were determined during earlier pilot simulations. The value of α was chosen from a log-uniform distribution since this parameter has an exponential effect on the behavior of v(t). The size of βwas limited, because at larger values, there would be no simulated vection dropouts (i.e., instead, the range of βwe chose allowed for possible perceptual alternations between vection ON and OFF periods).

### Results of the Virtual Experiment

**Figure 4.**
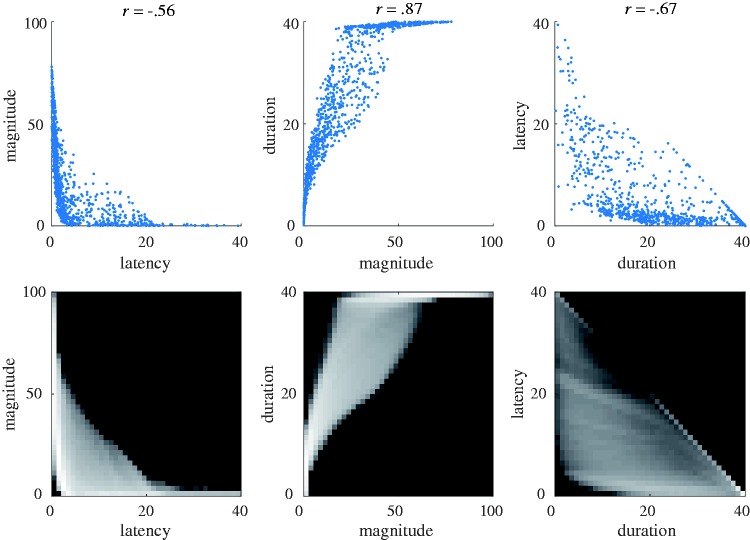
The top three panels show the relationships between the virtual vection onset, duration, and magnitude responses generated by OPVM (only the first 1,000 of the total 10,000 trials are shown here for the sake of visibility). Significance levels are all *p* < .001. The bottom three panels show their corresponding heat maps. Intensity indicates the density of data points (i.e., brighter cells include more data points).

The vection onset, duration, and magnitude data generated by OPVM for each of the 10,000 simulated trials is shown in Figure 4. These data are plotted as the correlations between (a) vection latency and magnitude, (b) vection duration and magnitude, and (c) vection latency and duration.

High correlations were found between all three of these simulated vection indices. In this analysis, “no vection” responses were treated as having a duration of 0 s and an onset latency of 40 s. In trials where vection was experienced, the sum of the onset and duration values was always less than 40 s (since there was always a finite delay before vection was experienced and motion stimulation only lasted 40 s). This is the reason that no data points appear in the upper right field of the latency-duration plot (Figure 4). Thus, these latency and duration data were not fully independent of each other (they were at least partially methodologically dependent on each other).

## OPVM Performance Compared With Laboratory Vection Data

To further test the model, we next compared the simulated vection data (discussed in the Testing OPVM section) with real vection data obtained in seven different laboratory experiments. This real data consisted of human vection onset latency, duration, and magnitude responses. The details of these laboratory experiments are described in the following subsections.

### Laboratory Experiments

Five out of these seven laboratory experiments had been published as scientific articles (in either English or Japanese—see Ogawa, Ito, & Seno, 2015; Ogawa & Seno, 2016; Ogawa, Seno, Matsumori, & Higuchi, 2015; Seno & Nagata, 2016; Seno, Ogawa, Tokunaga, & Kanaya, 2016). The remaining two experiments have yet to be published as papers. However, they have been both presented at international conferences (ICP: Ogawa, Seno, Ito, & Okajima, 2016; VSAC: Seno, Palmisano, & Nakamura, 2016).

#### Human participants

These experimental data were obtained from 107 different individuals (who were undergraduate students, graduate students, as well as staff and faculty members of Kyushu University). Participants reported no health issues at the time of testing. They had either normal or corrected to normal vision and no history of vestibular system diseases. While some of the authors of this article were participants, they did not know the purpose of these studies at the time of testing. Written informed consent was obtained from all participants prior to testing.

#### Apparatus

The vection stimuli were generated by and controlled via computers (MacBook Pro, MD101J/A, Apple Inc., Cupertino, CA; or ALIENWARE M18x, Dell Inc., Round Rock, TX) and presented on a 65-in. plasma display (3D VIERA TH-65AX800, Panasonic Corporation, Osaka, Japan) which had a resolution of 1920 × 1080 pixels and a refresh rate of 60 Hz. These experiments were all conducted in a dark room and participants always sat on a rocking chair to enhance their vection experience. No chin-rests or head-rests were used. Viewing distance to the display was held constant at approximately 57 cm across all of these experiments.

#### Stimuli

**Figure 5.**
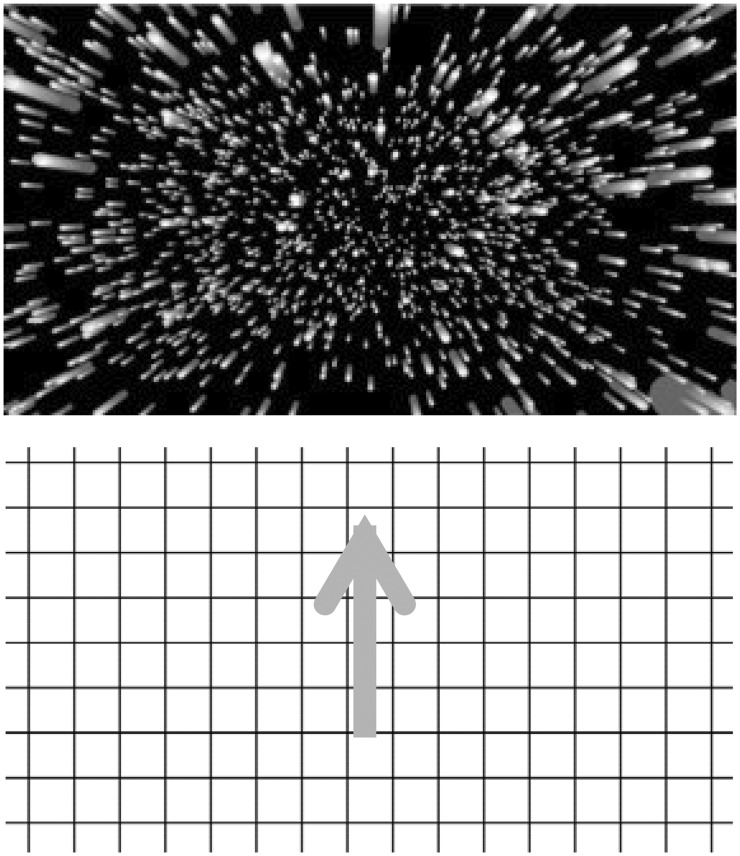
Schematic illustrations of the two types of stimuli used in the experiments in this article. (Top) Radially expanding optic flow. (Bottom) A vertically moving grid pattern.

Two different types of experimental stimulus displays were used. In some experiments, a radially expanding optic flow stimulus was used, whereas in the remainder, a vertical optic flow stimulus was used. In both cases, these visual motion displays subtended a visual area of 100 (horizontal) × 80 (vertical) degrees^2^ and the stimulus motion always lasted 40 s. The stimulus motion completely filled the display. Thus, the size of the stimulus and the display were approximately the same. The radially expanding pattern of optic flow consisted of white dots (38 cd/m^2^) presented on a black background (0 cd/m^2^). This display simulated forwards self-motion in depth at 16 m/s relative to a 3D cloud of 16,000 randomly positioned dots (see Figure 5, Top). As individual dots disappeared off the edges of the screen, they were moved back in depth to the far depth plane, thereby creating an endless optic flow display. Approximately 1,240 dots were visible in each frame, with each dot subtending a visual angle of 0.03° to 0.05° (their size remained constant as their simulated distances from the observer changed). Since these dots did not form a density gradient, motion perspective was the only cue to motion in depth. The second stimulus display presented the constant upward motion of a black grid (0 cd/m^2^) on a uniform white background (38 cd/m^2^)—it simulated downward self-motion at 18°/s (see Figure 5, Bottom). One side of each square in this rectangular grid subtended approximately 8° in visual angle.

#### Procedure

Participants observed these vection-inducing stimuli while sitting on a rocking chair inside a dark viewing chamber. Their task was (a) to press a button when they first experienced illusory self-motion and (b) to keep this button depressed as long as the experience continued (which provided data about both the onset latency and the duration of vection). After each stimulus presentation, they also had to report the subjective strength of their vection experience using 101-point rating scale (from 0 = *no vection* to 100 = *very strong vection*). Each stimulus display condition was repeated four times in each experiment. The 317 data sets used in this analyses were the result of testing 1,268 discrete vection trials. Each of the individual data sets consisted of the average onset, duration, and magnitude values obtained for a single subject in one experiment.

### Results

#### Laboratory vection data

**Figure 6.**
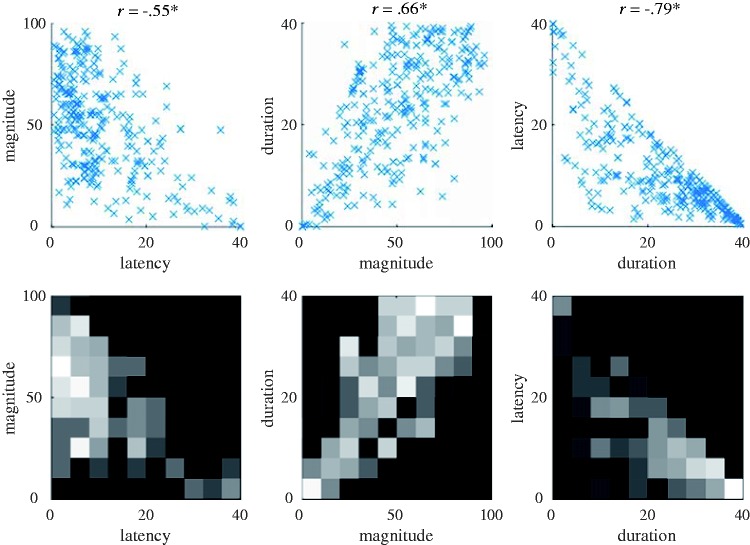
The top three panels show the vection onset, duration, and magnitude responses obtained in the seven laboratory experiments. The bottom three panels show their corresponding heat maps. Intensity again indicates the density of data points (i.e., brighter cells include more data points).

Correlational analyses were conducted on 317 discrete sets of laboratory-obtained vection data. Figure 6 shows the relationships between the three different vection measures. All combinations of these measures were found to generate significant correlations (latency–magnitude, *R* (317) = −.55, *p* < .001; duration–magnitude, *R* (317) = .66, *p* < .001; latency–duration, *R* (317) = −.79, *p* < .001; see also Figure 4). These three correlation coefficients were significantly different to each other (*z = *2.13, *p = *.03, *z = *3.51, *p < *.001, and *z = *3.51, *p < *.001, respectively, for latency-magnitude and duration-magnitude, duration-magnitude and latency-duration, and latency-magnitude and latency-duration). Magnitude ratings were found to account for 30% of the variability in vection onset latency and 44% of the variability in vection duration. The strongest relationship was found for the two time course measures—with vection duration accounting for ∼62% of the variability in vection onset latency responses. The strength of this relationship between vection onset and duration was presumably due in part to the unavoidable trade-off between the two time course measures (as vection onset latency increased, vection duration typically decreased. As noted earlier, these latency and duration data were at least partially methodologically dependent on each other).

#### Comparison of OPVM and laboratory results

**Figure 7.**
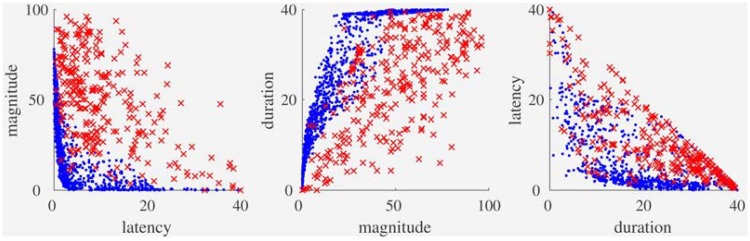
Comparisons of OPVM’s simulated data (blue) with the empirical data (red) obtained in the laboratory vection experiments.

When we compared the corresponding virtual and laboratory vection data with each other, we noticed a number of similarities in their distributions. To better visualize these similarities between OPVM-generated and human data, we superimposed data points from our earlier virtual (Figure 4, Top) and laboratory (Figure 6, Top) plots—thereby creating the new Figure 7.^6^

This new figure depicts the relationships between latency and magnitude, magnitude and duration, as well as latency and duration for the OPVM and laboratory-based vection response data. Both the OPVM and human data were found to produce (a) significant positive relationships between magnitude and duration (*R* = .87 and *R* = .66, respectively), (b) significant negative relationships between magnitude and latency (*R* = −.56 and *R* = −.55, respectively), and (c) significant negative relationships between latency and duration (*R* = −.67 and *R* = −.79).

OPVM also appeared to be successful in generating substantial variability in the responding. Indeed, this variability in responding appeared to mimic (at least superficially) some of the individual differences seen in the human responding. However, OPVM’s responding appeared to be less variable than the human responding. These discrepancies in response variability appeared to be more obvious in the latency versus magnitude and the duration versus magnitude plots (compared with the latency versus duration plot). These discrepancies will be discussed in detail later.

## Discussion

In this article, we developed and tested a model of responding to visually induced vection, the OPVM. OPVM was constructed based on three well-documented properties of the vection experience: (a) that there is a finite delay before the reported onset of vection, (b) that there is a subsequent increase of reported vection magnitude over time until vection responding eventually plateaus, and (c) that vection dropouts are reported to occur (after vection induction and before the display motion ceases). Next, in our 10,000 virtual trial simulation experiment, we attempted to model not only these three properties of vection but also commonly observed individual differences in vection responding (by altering the values of key parameters of OPVM: α, β, *T*, and θ). Vection onset latency, duration, and magnitude estimates were reconstructed (based on the OPVM response outputs) for each virtual trial. Finally, we compared the performance of our model with the results of previous laboratory studies which obtained the same vection measures. Statistical analyses of the real and model-based vection data indicated that all three measures correlated significantly with each other.

Our results demonstrate that both overall and specific vection responding (including individual differences) can be described quite well by OPVM. However, there also appeared to be some notable inconsistencies between the real and simulated vection response data. These can best be seen in the Latency-Magnitude correlation (Figures 7, Left) and the Duration-Magnitude correlation (Figure 7, Middle) plots. In the latter case, magnitude ratings appeared to be considerably larger for longer vection durations during simulation, whereas the equivalent relationship between magnitude and duration was noticeably weaker for the real vection data. We speculate that this particular discrepancy might reflect idiosyncrasies in human responding rather than potential inadequacies of OPVM. During the vection experiments, participants observed each visual motion display for 40 s and only provided their magnitude ratings after the display motion ceased. It is likely that the magnitude ratings made by our human participants did not accurately reflect the average strength of their vection experience across the entire trial but instead were biased by stronger vection experiences they had toward the end of the trial. If this explanation is valid, then this real versus simulated vection data discrepancy might reflect a recency effect.^7^ Future work should thus be aimed at incorporating such human response characteristics (particularly those common when making perceptual judgments) into OPVM.

To better understand and predict the conscious experience of vection, OPVM will need to be further developed and refined. In the current version of OPVM, we utilized sinusoidal and exponential functions in an attempt to model the experience of vection. However, these are rather simple mathematical functions. It is likely that more complex mathematical functions may be required to improve the model (e.g., it is highly likely that temporal changes in both human perception and responding may be different from the sinusoidal changes currently incorporated into OPVM). This will undoubtedly require further empirical investigations of vection (obtaining new data by using different display manipulations and other measurement methods). For example, several previous studies have had participants press different buttons that correspond to the subjective magnitude of the vection they are experiencing at the time (no vection, weak, modest, and strong) and then examined the total amount of time that each of these buttons was depressed during the trial (e.g., Mohler et al., 2005; Riecke et al., 2006, 2009, 2011; Seya, Shinoda, & Nakaura, 2015; Seya, Tsuji, & Shinoda, 2014; Seya, Yamaguchi, & Shinoda, 2015). The accumulation of these magnitude values over entire stimulus presentation period was then used to assess the overall vection experience. Other studies have used joysticks, slider devices, or levers to collect continuous ratings of vection magnitude over the entire course of each trial (e.g., Apthorp, Nagle, & Palmisano, 2014; Apthorp & Palmisano, 2014; Berthoz, Pavard, & Young, 1975; Bonato & Bubka, 2006; Bubka et al., 2008; Kim & Palmisano, 2010b; Palmisano, 2002; Seno, Yamada, & Palmisano, 2012; Telford et al., 1992; Trutoiu, Mohler, Schulte-Pelkum, & Bülthoff, 2009; Weech & Troje, 2017). By employing similar methods, we might be able to analyze the tendencies of the temporal change in vection strength more precisely and incorporate the results into OPVM. Vection strength and time averaging should therefore be further examined in future.

As noted earlier, another potential issue with the current investigation was that the latency and duration data (both reported in seconds) were not fully independent of each other. As in the majority of past laboratory studies, these data were at least partially methodologically dependent on each other (because the trial duration was fixed; therefore, longer vection onsets would be more likely to be associated with shorter vection durations—even factoring in the possibility of subsequent vection dropouts). An alternative way to examine the relationships between these temporal vection measures might be to recode the latter duration measure as a percentage of the time that vection was experienced as a function of the entire stimulus presentation period (as has been recently suggested by Keshavarz et al., 2017). For example, a vection experience lasting 30 s during a 40-s stimulus presentation period would be recoded as a % duration value of 75. Reexamining our model with this and other alternative vection response measures should therefore also be a future task for us.^8^

Furthermore, vection is not restricted to vision, but vection can also be induced by stimulating other sensory modalities, for example, auditory vection (e.g., Väljamäe & Sell, 2014; Väljamäe, 2009, for review) and cutaneous vection (Murata, Seno, Ozawa, & Ichihara, 2014). In the development of OPVM, only the properties of visually induced vection were considered. However, there are similarities between the vection experiences induced by visual and other perceptual modalities. Thus, it is possible that the model could be applied or extended to vection induced by other nonvisual modalities.

Although the OPVM has room for improvement as noted earlier, the current version is capable of describing the reported experience of vection quite well (despite its rather simplistic component functions). OPVM therefore has the potential to be a useful tool in understanding both the overall and specific experiences of vection.

## Notes

1 For example, Helmholtz (1867/1925) reported vection that he experienced while viewing a quickly moving river from above on a bridge.

2 Auditory (e.g., Keshavarz et al., 2014; Mursic, Riecke, Apthorp, & Palmisano, 2017; Väljamäe, 2009) and tactile (e.g., Murata et al., 2014; Nordahl, Nilsson, Turchet, & Serafin, 2012) motion stimulation have both been reported to produce similar (although often less compelling) illusions of self-motion in blindfolded observers. Illusory self-motion can also be induced by passively rotating the limbs of blindfolded observers (e.g., Howard, Zacher, & Allison, 1998) or having them step on a treadmill (e.g., Bles, 1981).

3 While these are the most common, other possible vection measures include the subjective speed of the self-motion (e.g., Apthorp & Palmisano, 2014; Brandt et al., 1973; de Graaf, Wertheim, & Bles, 1991; Kennedy et al., 1996; Palmisano, 2002; Sauvan & Bonnet, 1993, 1995; Telford & Frost, 1993; Young, Dichgans, Murphy, & Brandt, 1973) as well as changes in pupil dilation (e.g., Ihaya, Seno, & Yamada, 2014), eye movements (e.g., Brandt et al., 1974; Kim & Palmisano, 2008, 2010a, 2010b), and body sway (e.g., Apthorp, Nagle, & Palmisano, 2014; Palmisano, Pinniger, Ash, & Steele, 2009; Palmisano, Apthorp, Seno, & Stapley, 2014; Wei, Stevenson, & Körding, 2010). Nulling and other types of physical motions (e.g., Carpenter-Smith, Futamura, & Parker, 1995; Miller, O’Leary, Allen, & Crane, 2015; Nesti, Beykirch, Pretto, & Bülthoff, 2015; Palmisano & Gillam, 1998; Rosenblatt & Crane, 2015), nonvisual self-motion aftereffects (e.g., Cuturi & MacNeilage, 2014), and EEG (e.g., Palmisano, Barry, De Blasio, & Fogarty, 2016) have also been proposed to serve as vection measures.

4 The choice of which vection measures to include in such studies was likely made based primarily on practical reasons. It was often not possible to obtain all three vection measures together because of study design limitations.

5 Ratings are most commonly of the vection’s strength or magnitude (Brandt et al., 1973). However, ratings of its perceived speed (e.g., Kim & Palmisano, 2008), convincingness (e.g., Riecke et al., 2006), realism (e.g., Kruijff, Riecke, Trepkowski, & Kitson, 2015), degree of saturation (e.g., Allison, Howard, & Zacher, 1999; McAnally & Martin, 2008), and distance travelled (e.g., Palmisano, 2002) have also been obtained.

6 In terms of statistics, a similarity index is often used to compare multiple models (i.e., the comparisons between two or more models). Thus, in this study, it was difficult to show a certain similarity index between the raw data and simulated data. For this reason we provide qualitative, rather than quantitative, statements here.

7 In perceptual tasks, observers are able to judge the temporal average, but their judgments appear to be strongly dependent on local information around the time of stimulus offset (cf. VSS: Sato, Motoyoshi, & Sato, 2013).

8 We should state here again that latency and duration responses (both in seconds) were at least partially methodologically dependent. Even with this aspect, we still believe that the behaviors of all three (absolute) vection indices (onset, duration, and magnitude) are important for understanding the behaviors of the inner parameters underlying vection responding in the brain. These three indices are the most commonly obtained in human laboratory studies. In the past, the onset and duration vection measures have almost always been reported as absolute times in seconds. It is possible that these three measures used in the current investigation (onset, duration, and magnitude) might best represent different aspects of the overall vection experience (as suggested earlier by Palmisano & Chan, 2004). Consistent with this notion, experimental display manipulations are often reported to have inconsistent effects across these three different vection measures. For example, it is common for significant effects to be found between experimental and control conditions for vection magnitude, but not for onset latency and measures (e.g., Palmisano et al., 2011). This might suggest the need for a more comprehensive index of vection responding (e.g., Keshavarz et al.’s “% duration” index could be a possible candidate). In a number of recent studies, vection rating and time course measures have been collected simultaneously (instead of sequentially). Possibly, the increased use of such methodology might facilitate the creation a new and superior index of vection.
